# Pharmacologic Treatment Options for Insomnia in Patients with Schizophrenia

**DOI:** 10.3390/medicines5030088

**Published:** 2018-08-11

**Authors:** Lauren Stummer, Marija Markovic, Megan E. Maroney

**Affiliations:** 1 McLean Hospital, Belmont, MA 02478, USA; lauren.stummer@rwjbh.org; 2 Hackensack University Medical Center-Hackensack Meridian Health, Hackensack, NJ 07601, USA; marija.markovic@hackensackmeridian.org; 3 Ernest Mario School of Pharmacy at Rutgers, the State University of New Jersey, Piscataway, NJ 08854, USA; 4 Monmouth Medical Center-RWJBarnabas Health, Long Branch, NJ 07740, USA

**Keywords:** insomnia, schizophrenia, pharmacotherapy, paliperidone, melatonin, sodium oxybate, eszopiclone, antipsychotics

## Abstract

**Background:** Symptoms of sleep disorders, such as disturbances in sleep initiation and continuity, are commonly reported in patients with schizophrenia, especially in the acute phase of illness. Studies have shown that up to 80% of patients diagnosed with schizophrenia report symptoms of insomnia. Sleep disturbances have been shown to increase the risk of cognitive dysfunction and relapse in patients with schizophrenia. Currently, there are no medications approved specifically for the treatment of insomnia in patients with schizophrenia. **Methods:** A literature search was performed through OVID and PubMed to compile publications of pharmacotherapy options studied to treat insomnia in patients with schizophrenia. Articles were reviewed from 1 January 2000 through 1 March 2018 with some additional earlier articles selected if deemed by the authors to be particularly relevant. **Results:** Pharmacotherapies collected from the search results that were reviewed and evaluated included melatonin, eszopiclone, sodium oxybate, and antipsychotics. **Conclusions:** Overall, this review confirmed that there are a few evidence-based options to treat insomnia in patients with schizophrenia, including selecting a more sedating second-generation antipsychotic such as paliperidone, or adding melatonin or eszopiclone. Further randomized controlled trials are needed.

## 1. Introduction

Symptoms of sleep disorders, such as disturbances in sleep initiation and continuity, are commonly reported in patients with schizophrenia, especially in the acute phase of illness [1]. Insomnia is a particularly common condition with heterogeneous origin, which affects almost all individuals with psychosis at some point during their illness [2]. Studies have shown that up to 80% of patients diagnosed with schizophrenia report symptoms of insomnia [3]. Individuals with psychosis and co-occurring sleep-wake problems are more likely to have cognitive impairments, negative mood, reduced perceived ability to cope, daytime dysfunctions and an increased risk of suicide [2].

There is a complex relationship between insomnia, psychotic exacerbation, and antipsychotic treatment. A patient may experience sleeplessness due to psychosis-induced hypervigilance and fear, and sleep deprivation can aggravate or even precipitate a psychotic episode. As many as 40% of patients with schizophrenia relapse within one year despite maintenance treatment with an antipsychotic [4]. Previous research supports that a prodromal phase often precedes psychotic relapses in most patients with schizophrenia. Studies have shown that insomnia is ranked highly among the symptoms that appeared or worsened prior to a psychotic episode [5].

Despite its common occurrence, the treatment of insomnia in patients with schizophrenia is not well defined [6]. Studies have demonstrated that interventions that target insomnia symptoms can produce improvements in the severity of psychotic symptoms, quality of life, and functional outcomes [2]. Currently, there are no clear recommendations regarding the most effective treatment approach for insomnia in patients with schizophrenia. The aim of this review article is to assess the applicability of treatment options that have been studied for insomnia in patients with schizophrenia, along with utilizing sedating antipsychotics to combat insomnia. To our knowledge, this is the first comprehensive review of the literature to focus specifically on pharmacotherapies for insomnia in this population.

## 2. Materials and Methods

A literature search was performed through OVID and PubMed to compile publications on pharmacotherapy options studied to treat insomnia in patients with schizophrenia. All English-language articles published between 1 January 2000 through 1 March 2018, with some additional earlier articles selected if deemed by the authors to be particularly relevant, were reviewed. The reference sections of identified articles were also reviewed for publications that may have been missed by the initial search. Due to a relative paucity of available data, we chose not to exclude any clinical studies or case reports based on size or duration; however, no preclinical trials (animal data) were included. Publications that were not available in English were also excluded. Search terms used included: “insomnia in schizophrenia”, “treatment of insomnia in schizophrenia”, “sleep disturbances and schizophrenia” and “sleep and schizophrenia”. We reviewed and evaluated melatonin, eszopiclone, sodium oxybate, and antipsychotics, as the search results indicated that among available pharmacotherapy options for insomnia, these were the only agents specifically studied in patients with schizophrenia in the specified time frame. Benzodiazepines were used as a comparator group in two of the studies of zopiclone, but otherwise were not specifically evaluated. The impact of patient preferences on different treatment options for insomnia was also reviewed and summarized.

## 3. Results

### 3.1. Patient Preference of Treatment Options

Patient-centered care advocates for patients to be actively involved in making decisions about their treatment, but, according to research performed by Waters et al*.*, patients with schizophrenia and psychosis may feel unheard by mental health professionals during the decision-making process [2]. These authors completed a study with qualitative interview methodology to obtain data on the attitudes and preferences of people with schizophrenia and schizoaffective disorders toward three different types of therapies for insomnia: standard pharmacological, melatonin-based, and cognitive or behavior therapy [2].

Interviews regarding these three therapies discussed perceived advantages and limitations of each therapy, preferred approaches, factors that enabled patients to take up the preferred therapy, and personal strategies that have helped these individuals with sleep problems in the past. Patients’ perceived acceptability for pharmacotherapy was 22.5%, melatonin was 57.2%, and cognitive or behavioral therapies were 71.5%. Patients acknowledged the efficacy of pharmacologic treatment options, but still had concerns about drug interactions and potential side effects such as next-day drowsiness, poor attention, and concentration. Participants responded to the melatonin-based therapy approach more positively, but were concerned about how melatonin would interact with their current therapy. When discussing both melatonin-based and pharmacological therapies, patients perceived lack of control over the process as a limitation. Patients were more likely to be amenable to trying behavioral or cognitive therapies in order to have more control in their own treatment. Results from this study show the impact of including patients in the decision process when it comes to starting a new medication and the importance of patient education on various pharmacological treatments for insomnia [2].

### 3.2. Melatonin

Melatonin is an endogenous hormone secreted by the pineal gland that is linked to regulation of the circadian rhythm [7]. The synthesis and release of melatonin are stimulated by darkness and inhibited by light [8]. In humans, melatonin secretion increases soon after the onset of darkness, peaks in the middle of the night (between 02:00 and 04:00), and gradually falls during the second half of the night [7]. In a study completed by Ferrier et al*.*, melatonin was estimated in serum samples taken from patients diagnosed with schizophrenia and a control group at 24:00 and 08:00 with the goal to determine the relationship between melatonin secretion and psychiatric diagnosis and to examine factors which could account for any variations. Melatonin at 24:00 was significantly higher (*p* < 0.05) in the control group when compared to the group with schizophrenia. There was no statistically significant difference in melatonin levels at 08:00 between the two groups. The mean 24:00 to 08:00 ratio was 3.0 for the control group and 1.7 for the schizophrenia group; which was statistically significant (*p* < 0.05). When the effect of body weight was taken into consideration (the average body weight of the control group was significantly greater than the schizophrenia group) using an analysis of co-variance, the difference between the patients with schizophrenia and the control group was no longer statistically significant (*p* < 0.2) [9].

Meta-analyses suggest that melatonin conveys little to no benefit in patients with sleep disorders [8,10]; however, these meta-analyses only included one study that examined the usefulness of melatonin specifically in schizophrenia. As discussed by Ferrier et al., schizophrenia is a condition that may disturb patterns of melatonin secretion, leading to insomnia. The superseding studies obtained data for the use of melatonin specifically in patients with schizophrenia [9].

In a randomized, double-blind, cross-over study completed by Shamir et al*.*, patients with chronic schizophrenia were given either 2 mg of controlled-release melatonin or placebo. The melatonin or placebo was taken two hours before desired bedtime for a period of three weeks, with a one-week placebo washout period between the two treatment periods. Endogenous melatonin production was measured using urine samples from each patient, which were collected every three hours between 21:00 and 09:00. Actigraphy was performed for three consecutive nights at the end of each period. Sleep parameters based on activity and rest parameters were compared for the whole patient population to compare the efficacy of melatonin to placebo. A separate analysis was performed for patients subdivided into high versus low sleep efficiency. All patients (n = 19) who participated in the study had low melatonin output when compared to the reported amount excreted at night by healthy young or elderly patients with good sleep quality. The values of various sleep parameters determined by actigraphy at the end of melatonin and placebo treatment periods were not statistically significantly different. Melatonin replacement significantly improved rest-derived sleep efficiency compared with placebo (83.5% vs. 78.2%, *p* = 0.038) in this population. Improvement of sleep efficiency was significantly greater (*p* < 0.0014) in patients with lower than average sleep efficiency (80% vs. 67%) than in patients with high sleep efficiency (88% vs. 90%). In addition, during melatonin therapy, sleep latency was decreased by 40 min and sleep duration was increased by 45 min in low-efficiency sleepers, but neither of these endpoints reached statistical significance. In high-efficiency sleepers, sleep latency and total sleep time were minimally changed and not statistically different [1].

In a double-blind, placebo-controlled study completed by Kumar et al, the effectiveness of melatonin was assessed in patients with schizophrenia who were being treated as outpatients. A total of 40 patients were included in this study. Twenty patients were randomized into flexibly dosed melatonin (3–12 mg/night) and 20 were randomized to placebo. Assessments of aspects of sleep functioning were obtained by a patient completed questionnaire which was completed daily for 15 days after the initiation of melatonin or placebo. The modal stable dose of melatonin was 3 mg. At endpoint, there was no statistically significant difference between the melatonin and placebo groups for time to fall asleep (melatonin 1.14 h vs. 1.11 hours, *p* = 0.38). The melatonin-treated patients showed a significantly greater reduction in the number of nighttime awakenings (0.75 vs. 1.70, *p* = 0.045) and slept for longer than did the placebo-treated patients (5.7 hours vs. 5.4 hours, *p* = 0.021). Compared to placebo, the melatonin group had significant improvements in multiple factors related to sleep, such as morning freshness, morning headache, morning dullness, daytime mood, and daytime levels of functioning, while not causing next-day sedation [11].

A study completed by Romo-Nava et al*.* further investigated the positive outcomes of melatonin, specifically on metabolic parameters in patients taking second-generation antipsychotics. This study was an eight-week, double-blind, randomized, placebo-controlled, parallel-group clinical trial including patients with schizophrenia and bipolar disorder. Outcomes measured included weight, blood pressure, lipid monitoring, glucose monitoring, body composition, and anthropometric measures. Of the 44 patients treated with second-generation antipsychotics, 20 patients were randomized to receive melatonin 5 mg and 24 patients were randomized to placebo. Thirteen patients (54.2%) in the placebo group and 15 patients (75%) in the melatonin group were being treated with medium-risk of metabolic effects second-generation antipsychotics, while 11 (45.8%) and five (25%) patients received high-risk second-generation antipsychotics, respectively. The melatonin group showed a significant decrease in diastolic blood pressure (5.1 vs. 1.1 mmHg for placebo, *p* = 0.003) and gained less weight than the placebo group (1.5 vs. 2.2 kg, *p* = 0.040). Effects on fat mass change (0.2 kg for melatonin vs. 2.7 kg with placebo *p* = 0.032) and diastolic blood pressure (5.7 vs. 5.5 mmHg, respectively, *p* = 0.001) were only observed in the bipolar disorder and not in the schizophrenia group. No adverse events were reported [12].

Melatonin appears to be well tolerated in patients with schizophrenia and has shown benefit in improving sleep parameters as well as protection against the adverse metabolic effects of second-generation antipsychotics.

### 3.3. Sodium Oxybate

Gamma-aminobutyric acid (GABA)-B receptor agonists have been shown in rats to increase slow-wave sleep, which is a sleep parameter that is particularly impacted in patients with schizophrenia. Gamma-hydroxybutyric acid (GHB) is converted to GABA and subsequently acts as an agonist at GABA-B receptors. GHB has been studied in open-label and controlled trials of patients with schizophrenia. One open-label trial of 1–8 g/day of GHB showed that 60%–70% of patients experienced improvement in symptoms, especially in those patients with insomnia or negative symptoms. Two additional controlled trials did not show an overall benefit in schizophrenia symptoms. These trials did not include objective or subjective measures of sleep [13].

A subsequent trial of sodium oxybate, the sodium salt of GHB, was performed to specifically examine its impact on insomnia in patients with schizophrenia. Eight patients (seven male and one female) who were on a stable dose of antipsychotic for at least three weeks were included in this open-label trial. Patients were excluded if they had sleep apnea, a diagnosis of substance dependence in the past three months, a history of alcohol dependence or persistent need for sedative-hypnotic treatment. The average baseline Positive and Negative Symptom Scale (PANSS) score was 89.9, meaning they were moderately to markedly ill, and the average Pittsburgh Sleep Quality Index (PSQI) score was 11.3, indicating significant sleep disturbance. Included patients were tapered off their current sedative-hypnotic medication and placed on sodium oxybate 4.5 g/night titrated up to 9 g/night for a total of four weeks. Subjective sleep quality was measured using the PSQI and the Epworth Sleepiness Scale (ESS). The secondary outcomes were changes in the PANSS and cognitive function as measured by the Measurement and Treatment Research to Improve Cognition in Schizophrenia (MATRICS) battery. Significant improvement was seen not only in the PSQI (*p* = 0.002) and ESS (*p* = 0.02), but also in the PANSS total and PANSS negative symptoms scores (*p* = 0.04 for both). No difference was seen in the MATRICS. Polysomnography also showed a significant increase in slow-wave sleep and a decrease in rapid eye movement (REM) sleep. Mild nausea and nocturnal enuresis occurred in two patients each [14].

The potential benefit of sodium oxybate needs to be weighed against its significant risks including overdose, withdrawal and abuse, particularly in patients with a history of substance abuse [15].

### 3.4. Zopiclone/Eszopiclone

Traditional sedative-hypnotic medications such as benzodiazepines are frequently used to treat insomnia and manage symptoms associated with schizophrenia such as agitation. However, benzodiazepines have been found to impair slow-wave sleep and REM sleep, sleep parameters which may already be impaired in patients with schizophrenia. Conversely, non-benzodiazepine sedative hypnotics such as zopiclone have been reported to enhance slow-wave sleep with minimal effects on REM sleep, although in a small study of six patients with schizophrenia there were minimal differences in polysomnographic (PSG) measures when patients were switched from benzodiazepines to zopiclone. In this study of patients who were being treated with benzodiazepines for at least eight weeks, soundness of sleep improved when patients were switched to treatment with zopiclone for eight weeks. Symptoms of anxiety, emotional withdrawal, total Brief Psychiatric Rating Scale scores and negative symptoms scores also improved over the eight-week period. One patient developed insomnia after the switch to zopiclone, which improved with an increase in zopiclone dose, and one patient complained of bitter taste in the mouth, which is a common adverse effect of zopiclone [16,17].

Eszopiclone, a stereoisomer of zopiclone, has also been investigated for its potential use in insomnia in schizophrenia patients. Eszopiclone’s effects on sleep parameters and learning were first studied in a double-blind placebo-controlled trial of 21 patients with schizophrenia without insomnia. Patients were excluded if they had a diagnosed sleep disorder, a history of treatment with sleep medications or a history of substance abuse. Ten patients were randomized to receive eszopiclone 3 mg and 11 to placebo for a period of two consecutive nights. Patients treated with eszopiclone had a significant increase in sleep spindles during Stage 2 sleep as compared to placebo, which could theoretically improve memory consolidation. There were no other significant differences in sleep quality or architecture between the two groups [18].

Another controlled trial in 39 patients randomized subjects to either 3 mg of eszopiclone or placebo and compared changes in the Insomnia Severity Index (ISI), MATRICS, sleep diary entries, psychiatric symptoms and quality of life over eight weeks. Patients were included if they had a diagnosis of stable schizophrenia or schizoaffective disorder and sleep difficulties at least twice per week with an ISI score of at least 10, indicating that they had at least subthreshold insomnia symptoms. Patients were excluded if they had a diagnosis of alcohol or substance dependence, a medical or psychiatric disorder that could impair sleep, or if they were on a medication (other than an antipsychotic) that could affect sleep. Patients treated with eszopiclone were started on 2 mg daily at bedtime for the first week before increasing to the 3 mg dose. Thirty-six patients received study medication and were included in the final analysis, 19 in the eszopiclone arm and 17 in the placebo arm. At baseline, patients in the eszopiclone arm did have significantly worse scores in the general feeling upon wakening item of the ISI. At the end of the trial, the eszopiclone group showed significantly more improvement in the ISI total score than the placebo group (*p* = 0.039), as well as on the general feeling upon awakening (*p* = 0.013) and daytime fatigue (*p* = 0.025) items specifically. There were no differences between the eszopiclone treated patients and the placebo-treated patients in sleep diary items, clinical measures, quality of life or cognitive functioning. Adverse events seen in the trial were mild and included unpleasant/metallic taste, sedation, dry mouth and headache [6].

Based on its efficacy data for insomnia in the general population and the data summarized above, eszopiclone may be considered a reasonable treatment option for insomnia in patients with schizophrenia.

### 3.5. Antipsychotics

Treatment of schizophrenia patients with either first- or second-generation antipsychotics is associated with an overall improvement in sleep quality and efficiency, either by relief of psychosis or as an effect of the drug on various neurotransmitter receptors such as acetylcholine, dopamine, histamine, norepinephrine, and serotonin [19]. Blockade of histamine receptors promotes a sedative, sleep-enhancing effect. Anticholinergic effects can decrease the intensity of REM sleep and lengthen REM latency. Serotonin receptor antagonism promotes sedation and may increase the amount of slow-wave sleep, whereas agonism at 5-HT_1A_ receptors may cause sleep disturbances and suppress REM sleep. Alpha-1 adrenergic receptor inhibition appears to promote a sleep-enhancing effect and may increase REM sleep. Antagonism of dopamine receptors can result in restless leg syndrome, which may interfere with sleep [19]. As such, the variability in efficacy of the antipsychotic on sleep structure appears to be related to the individual receptor binding profiles of the drug for the aforementioned neurotransmitters. Frequently, the use of more sedating second-generation antipsychotics such as quetiapine, olanzapine, or risperidone is utilized in schizophrenia patients complaining of insomnia symptoms [19].

Overall, switching from a first-generation antipsychotic to a second-generation antipsychotic has been shown to improve subjective sleep quality, and this improvement in sleep quality tends to correlate with an improvement in negative symptoms [20]. This improvement may be due to differential effects on slow-wave sleep. Yamashita et al. evaluated the effect on subjective sleep quality, as measured by the PSQI, of switching from first-generation (typical) antipsychotics to second-generation (atypical) antipsychotics in 92 inpatients with schizophrenia over a study period of eight weeks [21]. The mean number of typical antipsychotics being administered at baseline was 2.2, and the most common drugs were haloperidol (70%), chlorpromazine (42%), and levomepromazine (25%). Patients were cross-tapered from their typical antipsychotic regimen over four weeks to one of four atypical antipsychotics: olanzapine (n = 20), quetiapine (n = 28), risperidone (n = 20), or perospirone (n = 24). Subjective sleep quality was evaluated by the PSQI at baseline and eight weeks after the switch was complete. At baseline, the mean PSQI total score was 8.58, indicating a poor quality of sleep. After eight weeks of atypical antipsychotic dosing, the PSQI total score was statistically significantly decreased (7.20, *p* = 0.002), indicating improvement in sleep quality. Subjective sleep quality, sleep latency, sleep efficiency, sleep disturbances, and daytime dysfunction sub-scores also improved statistically significantly (*p* < 0.05 for all). Sleep duration and use of sleep medication remained unchanged. Of note, olanzapine, risperidone, and quetiapine were shown to be superior to perospirone in their improvement in PSQI total score, sleep duration, sleep efficiency, and daytime dysfunction [21].

Yamashita et al. also compared the impact of risperidone versus haloperidol on sleep structure as measured by polysomnogram over the course of one night in ten schizophrenia patients without current or recent sleep disturbance [22]. The duration of slow-wave sleep was significantly longer in the risperidone group compared to the haloperidol group (*p* = 0.028). However, there were no significant differences between the two groups in terms of sleep latency, total sleep time, or sleep efficiency in this population.

Dursun et al*.* investigated the effect of risperidone as compared to first-generation antipsychotics (chlorpromazine, haloperidol, or flupenthixol) on sleep quality and continuity in 16 treatment-responsive schizophrenia patients with minimal residual psychotic features in an outpatient prospective pilot study [23]. They also included a sex- and age-matched control group consisting of eight healthy patients. Continuity of sleep was evaluated using at-home wrist actigraphy worn by patients for five consecutive nights and sleep quality was patient evaluated using a visual analog scale. Prior to study initiation, all patients in the two antipsychotic groups received at least 20 weeks of treatment with the same antipsychotic they received during the study. Patients in the risperidone group received a mean daily dose of 9.5 mg risperidone for 43 weeks, and patients in the first-generation antipsychotic group received a mean daily dose of 606 mg (chlorpromazine equivalent) for 191 weeks. The data showed that compared to the healthy control group, patients taking first-generation antipsychotics had significantly lower sleep quality, more morning sleepiness and a significantly higher movement index during sleep. Patients in the risperidone group did not differ significantly from the control group in sleep quality or morning sleepiness, but did have a higher movement index [23].

Maixner et al*.* studied the effect of haloperidol or thiothixene on polysomnographic measures in 14 schizophrenia patients hospitalized for an acute psychotic exacerbation [24]. Study subjects were medication free for a minimum of two weeks before initial sleep electroencephalogram (EEG) recordings, and three subjects were psychotropic naïve. Polysomnography was measured for two nights pre-treatment and for two nights after about 23 days of antipsychotic treatment with clinically determined doses of either haloperidol or thiothixene. After 3–4 weeks of treatment, sleep latency decreased (62.3 min at baseline vs. 29 min after treatment, *p* = 0.03), total sleep time increased (330.1 min at baseline vs. 385.9 min after treatment, *p* = 0.005), and sleep efficiency increased (79.1% at baseline vs. 87.9% after treatment, *p* = 0.03) [24].

Kluge et al*.* (2014) conducted a randomized, double-blind, single-center study comparing the effects of clozapine and olanzapine on sleep patterns and occurrence of restless leg syndrome (RLS) in 30 patients with schizophrenia, schizophreniform disorder, or schizoaffective disorder [25]. After an initial washout period, patients were randomized to receive either olanzapine 5–25 mg daily or clozapine 100–400 mg daily over a study period of six weeks. The modal dose of olanzapine and clozapine received in either group was 15 mg and 200 mg, respectively. The data showed that, compared to baseline, treatment with either olanzapine or clozapine increased total sleep time, sleep efficiency, and decreased sleep onset latency. Time spent in REM sleep increased from baseline in both groups, but the increase was significantly greater in the olanzapine group. Interestingly, olanzapine caused an 80% increase from baseline in slow-wave sleep, compared to clozapine, which caused a 6% decrease. RLS occurred only transiently in either treatment group [25].

Lee et al*.* investigated the effects of clozapine therapy on nocturnal growth hormone secretion and sleep architecture in five male schizophrenia inpatients compared with five healthy age- and sex-matched volunteers during an early therapy phase (within one week) and prolonged therapy (6–8 weeks) [26]. The mean daily dose of clozapine during the prolonged therapy phase was 290 mg. Sleep was measured using laboratory-based polysomnographic examinations. Patients with schizophrenia had significant improvement in sleep efficiency and total sleep time from baseline to the early therapy phase, without much further change during the prolonged therapy phase [26].

Muller et al*.* (2004) evaluated the effect of olanzapine on sleep in a four-week prospective open-label polysomnographic study in ten male patients with schizophrenia exhibiting predominantly negative symptoms [27]. Included subjects had never taken olanzapine before, and received 15 or 20 mg of olanzapine daily during the study. Compared to baseline, treatment with olanzapine significantly increased sleep period time and total sleep time, and significantly decreased sleep onset latency (*p* < 0.05 for all three parameters) [27].

The only antipsychotic to be investigated for the treatment of insomnia in patients with schizophrenia in a randomized, placebo-controlled fashion is paliperidone. Luthringer et al*.* conducted a multicenter, double-blind, randomized, placebo-controlled trial comparing paliperidone extended release (ER) 9 mg tablets with placebo over an active treatment period of 14 days in 42 participants with symptomatically stable schizophrenia and schizophrenia-related insomnia [28]. Compared to the placebo group, patients randomized to the paliperidone ER group experienced several statistically significantly improved aspects of sleep architecture and continuity, including increased total sleep time and decreased latency to sleep onset, as measured by polysomnogram. Study participants also evaluated their sleep quality daily using the Leeds Sleep Evaluation Questionnaire. The between-group differences were not statistically significant for the subjectively assessed variables, which included getting to sleep, quality of sleep, ease of awakening, and behavior following awakening [28].

Kongsakon et al*.* conducted an open-label prospective study evaluating the effect of six months of treatment with flexibly dosed paliperidone ER (3–12 mg daily) on sleep quality and daytime drowsiness in 984 Southeast Asian patients with schizophrenia [29]. At baseline, the mean PANSS score was 74, indicating a moderately ill population. The study population had previously unsuccessful treatment with other oral second-generation antipsychotics due to treatment failure or tolerability issues prior to entering the study. Sleep quality and daytime drowsiness were assessed using visual analog scales scored from 0–100, with higher scores indicating improved sleep quality on the sleep quality scale and more daytime drowsiness on the daytime drowsiness scale. The data found that from baseline to the end of the study, paliperidone ER statistically significantly improved sleep quality (76.44 vs. 65.48, *p* < 0.001) and decreased daytime drowsiness (23.18 vs. 34.22, *p* < 0.001) [29].

Antipsychotics appear to have a variable effect on sleep in patients with schizophrenia. Paliperidone in particular appears to be an effective treatment option for insomnia in schizophrenia.

### 3.6. Polypharmacy

There is a paucity of clinical data on the use of antipsychotic polypharmacy for the treatment of sleep symptoms in schizophrenia, and evidence is largely practice-based. The augmentation of an antipsychotic regimen with low-dose immediate-release quetiapine (25–75 mg nightly) is a common practice in many patients with psychiatric disorders [30,31,32]. The sedative properties of quetiapine can be attributed to its strong antagonism at histamine H_1_ receptors and moderate antagonism at serotonin type 2A (5-HT_2A_) receptors [33]. It is important to note that even at low doses, quetiapine has been associated with significant increases in weight [33,34].

Hanisch et al*.* reported a case of improvement in treatment refractory insomnia with the addition of quetiapine 300 mg nightly to sertindole 20 mg (a second-generation antipsychotic) in a 46-year old male with paranoid schizophrenia [35]. The patient had recently discontinued clozapine therapy due to the development of a metabolic syndrome and increased hallucinations and delusions of persecution. Prior to the initiation of quetiapine, the patient had trials of zopiclone, zolpidem, and chloral hydrate for insomnia, which only led to short-term improvement in subjective sleep quality. Within a period of two months, add-on therapy with quetiapine resulted in improved subjective quality of sleep regarding sleep latency and number of nocturnal awakenings. After four months of quetiapine, sleep latency further improved (less than one hour) and the patient described his quality of sleep as mostly restorative, spending eight hours in bed as compared to four hours prior to the addition of quetiapine. In this case report, the author notes that the combination of two antipsychotics did not result in increased QTc intervals or metabolic side effects [35].

Waters et al*.* surveyed the effects of polypharmacy on subjective sleep quality using the PSQI in 83 long-stay psychiatric patients diagnosed with schizophrenia or bipolar disorder [36]. Seventy percent of respondents scored greater than 5 in global PSQI scores, indicating a population with significant sleep disturbances. All patients took antipsychotics, with the number of concurrent antipsychotics per person ranging between one and four. The results of the questionnaire indicated that there was a correlation between an increasing number of antipsychotics, as well as an increasing total dosage, and decreased sleep dysfunction. However, the authors noted that antipsychotics only explained a small proportion of variance in subjective sleep quality, suggesting that antipsychotics marginally improve but do not necessarily normalize sleep patterns [36].

## 4. Conclusions

Since current evidence shows that poor sleep is something that affects the majority of individuals with schizophrenia at some point during their illness and that it may act as a barrier to recovery, it is a clinical priority to fully address insomnia in people with schizophrenia. It is important for participants to be included in making choices regarding the type of medication therapy selected. Overall, this review confirmed that there are few evidence-based pharmacologic options to treat insomnia in patients with schizophrenia. The most rigorously studied pharmacotherapies include paliperidone, melatonin and eszopiclone.

There are several limitations to this review. Many of the trials included had a very small sample size, thereby limiting the power and applicability of the results to the general population of patients with schizophrenia. We were unable to include non-English publications, eliminating potentially relevant publications. We also chose to limit our search to include articles published after 1 January 2000. Although we included some earlier published trials based on our review of relevant references, we may have omitted some earlier publications on benzodiazepines and barbiturates, for example. As several of the medications in these pharmacologic categories are no longer preferred first-line treatments for insomnia in general, due in part to concerns of abuse and respiratory depression, particularly in overdose, they should not be used preferentially in this population either [37]. Non-pharmacologic treatments, while critically important for treating insomnia, are outside the scope of this review.

Very few studies of sedating antipsychotics were identified in the literature. This is surprising given the theoretical convenience of using a medication that is already indicated for use in schizophrenia. Quetiapine, in particular, is commonly used off-label to treat insomnia in patients with mental health disorders, but evidence supporting this strategy is lacking [33]. Out of the currently available antipsychotics, only paliperidone has been studied in a randomized, controlled setting for insomnia in patients with schizophrenia.

Further randomized controlled trials are needed to establish the most appropriate pharmacotherapy for insomnia in schizophrenia. Many clinical trials exclude patients with psychiatric illness such as schizophrenia. Pharmacotherapies studied more thoroughly for insomnia in this population, such as melatonin and paliperidone, are not recommended treatments for primary insomnia in the general population [37]. Of the pharmacotherapies reviewed here, only eszopiclone is recommended by the American Academy of Sleep Medicine guidelines for the treatment of primary insomnia [37]. Other recommended treatments for primary insomnia such as zaleplon, zolpidem, ramelteon, doxepin and suvorexant have not been studied in the schizophrenia population.

Based on the available literature, a reasonable approach to treating insomnia in a patient with schizophrenia could include switching their antipsychotic to paliperidone, or adding eszopiclone or melatonin. If these methods are ineffective, cautiously adding sodium oxybate could be recommended. Research priorities include studying the effects of more sedating antipsychotics and guideline-supported insomnia medications such as zaleplon, zolpidem, ramelteon, doxepin and suvorexant in patients with insomnia and schizophrenia.
